# Association between lipid accumulation product and psoriasis among adults: a nationally representative cross-sectional study

**DOI:** 10.1186/s12944-024-02123-y

**Published:** 2024-05-17

**Authors:** Caiyun Zhang, Xiaoping Dong, Jun Chen, Fang Liu

**Affiliations:** 1 Department of Medical Cosmetology, Nanjing Hospital of Chinese Medicine Affiliated to Nanjing University of Chinese Medicine, Nanjing, 210022 China; 2 Department of Dermatology, Jinling Hospital, Affiliated Hospital of Medical School, Nanjing University, Nanjing, 210002 China; 3 Department of Dermatology, The Fourth Affiliated Hospital of Nanjing Medical University, Nanjing, 210031 China

**Keywords:** NHANES, Psoriasis, Lipid accumulation product, Obesity

## Abstract

**Background:**

Lipid accumulation product (LAP) is an accessible and relatively comprehensive assessment of obesity that represents both anatomical and physiological lipid accumulation. Obesity and psoriasis are potentially related, according to previous research. Investigating the relationship between adult psoriasis and the LAP index was the goal of this study.

**Methods:**

This is a cross-sectional study based on data from the National Health and Nutrition Examination Survey (NHANES) 2003–2006 and 2009–2014. The association between LAP and psoriasis was examined using multivariate logistic regression and smoothed curve fitting. To verify whether this relationship was stable across populations, subgroup analyses and interaction tests were performed.

**Results:**

The LAP index showed a positive correlation with psoriasis in 9,781 adult participants who were 20 years of age or older. A 27% elevated probability of psoriasis was linked to every unit increase in ln LAP in the fully adjusted model (Model 3: OR 1.27, 95% CI 1.06–1.52). In comparison with participants in the lowest ln LAP quartile, those in the highest quartile had an 83% greater likelihood of psoriasis (Model 3: OR 1.83, 95% CI 1.08–3.11). This positive correlation was more pronounced for young males, participants who had never smoked, non-drinkers, participants who exercised little, as well as non-hypertensive and non-diabetic participants.

**Conclusions:**

This study found that the LAP index and adult psoriasis were positively correlated, especially in young males without comorbidities. Therefore, it is proposed that LAP may serve as a biomarker for early diagnosis of psoriasis and tracking the effectiveness of treatment.

**Supplementary Information:**

The online version contains supplementary material available at 10.1186/s12944-024-02123-y.

## Background

Approximately 3.0% of the United States (U.S.) adult population suffers from psoriasis, a prevalent immune-mediated disease [1]. The incidence of psoriasis is comparable in women and men. The two peak age ranges for psoriasis are 18 to 39 and 50 to 69 years, although it can develop at any age [2]. It is well known that psoriasis not only causes extensive, recurrent patches and plaques of the skin but may also be accompanied by multiple metabolism-related comorbidities, such as obesity, hypertension, diabetes, dyslipidemia, non-alcoholic fatty liver disease (NAFLD) and metabolic syndrome (MetS) [3, 4]. Psoriasis has resulted in a significant socioeconomic burden due to its incurability and potential involvement of multiple organ systems [5, 6].

The prevalence of obesity has steadily climbed globally over the past few years, posing a serious threat to health [7]. Obesity is strongly linked to the prevalence of metabolism-related diseases, such as type 2 diabetes, hypertension, obstructive sleep apnoea, NAFLD and cardiovascular disease [8]. Currently, the commonly used clinical indicators for obesity assessment include the body mass index (BMI) and waist circumference (WC). BMI represents overall obesity but cannot assess different body components, such as bone density, muscle mass and distribution of fat, so some researchers believe that it is a relatively crude and controversial approach for assessing the risk of some diseases and mortality [9–11]. WC is used to assess the degree of abdominal obesity (or central obesity), which causes metabolic disorders that can lead to diabetes, NAFLD, MetS and cardiovascular disease [8, 12]. However, WC cannot distinguish between visceral and subcutaneous adipose tissue, and the former indicates ectopic fat accumulation, which causes organ dysfunction and insulin resistance [8].

In another approach, lipid accumulation product (LAP) uses WC and fasting triglycerides (TG) concentration to characterize lipid overaccumulation, which can reflect the combined anatomic and physiologic changes in adults [13]. Several studies have suggested that LAP could be a potential indicator for cardiovascular disease, type 2 diabetes, insulin resistance, NAFLD and MetS [13–16].

According to two single-center case-control studies conducted to date, psoriasis patients had a considerably higher LAP index than healthy individuals [17, 18]. However, no prior research has explored the connection between LAP and psoriasis in a population that is nationally representative. The main objective of this study was to explore whether LAP and the prevalence of psoriasis are related, using the 2003–2006 and 2009–2014 National Health and Nutrition Examination Survey (NHANES) datasets. It is hypothesized that increased LAP scores may be associated with a high prevalence of psoriasis. By revealing a link between lipid metabolism, obesity and psoriasis, this study will help in the understanding of psoriasis as a systemic metabolic disorder and its association with other metabolism-related diseases. In clinical practice, LAP may be potentially valuable for psoriasis diagnosis and chronic disease management.

## Materials and methods

### Data sources and study population

NHANES, which polls approximately 5,000 individuals annually from all over the U.S., provided the data for this study. This project is performed by the National Center for Health Statistics (NCHS) to evaluate the nutritional status and general health of the non-institutionalized U.S. population. Because the study design employs a stratified multistage probability sampling process, it is highly representative [19]. The whole datasets are publicly and freely accessible at https://www.cdc.gov/nchs/nhanes/.

**Fig. 1:**
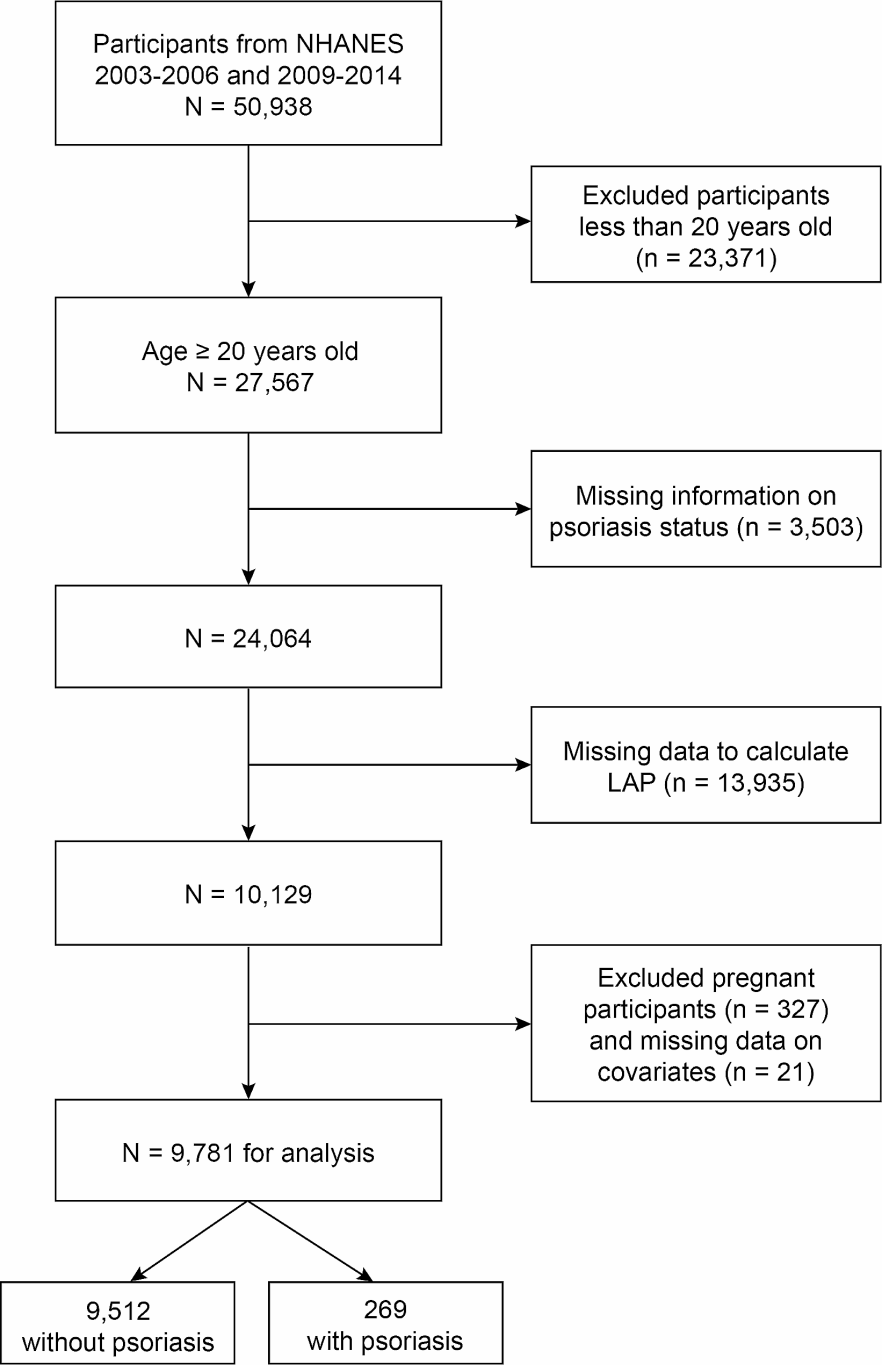
Flow chart of participant selection. Abbreviations: NHANES, National Health and Nutrition Examination Survey; LAP, lipid accumulation product

Data for the present study were obtained from five NHANES survey cycles, from 2003 to 2006 and 2009 to 2014, since only these cycles contained data on both psoriasis status and LAP. The following is a list of the exclusion criteria: (1) participants younger than 20 years of age; (2) participants with missing information to define psoriasis; (3) participants with incomplete data to calculate LAP; (4) pregnant women; (5) participants with missing data on necessary covariates (including 10 on education level, 4 on marital status, 5 on smoking status, 2 on blood pressure information). Ultimately, this study comprised 9,781 participants in total (Fig. 1).

### Exposure and outcome definitions

In NHANES, anthropometric assessments are carried out by trained health technicians at the mobile examination center. Specifically, WC is measured with a tape measure at the upper edge of the iliac crest in centimeters (cm). The following formulas are used to calculate the LAP score: [WC (cm) – 65] × [TG (mmol/L)] for men and [WC (cm) – 58] × [TG (mmol/L)] for women. According to the original protocol, men with a WC of 65 cm or less, women with a WC of 58 cm or less, and all participants with serum TG concentrations > 15 mmol/L were excluded [13].

Psoriasis diagnosis was via a self-reported questionnaire, which for different cycles was the dermatology questionnaire or medical conditions questionnaire. Specifically, participants were asked that “Have you ever been told by a doctor or other health care professional that you had psoriasis?” The reply options were “Yes” and “No”. The reliability of self-reported psoriasis conditions has been supported by previous research [20].

### Covariates

The present study considered the following covariates that could have an impact on psoriasis: age (years), sex (male/female), race (Mexican American, other Hispanic, non-Hispanic White, non-Hispanic Black and other race), education level (less than high school, high school and more than high school), marital status (cohabitation/solitude), poverty-to-income ratio (PIR), smoking status, drinking status, physical activity, total cholesterol (TC, mmol/L), hypertension, diabetes and medication status. For subgroup analysis, three age groups were used to classify the study population: < 40 years, 40 ≤ age < 59 years and ≥ 60 years. Smoking status was grouped as never smoked (< 100 cigarettes in life), former smoker (≥ 100 cigarettes but has quit) and current smoker (≥ 100 cigarettes and currently smoking). Alcohol intake status was classified by the question “Had at least 12 alcohol drinks/1 yr?”. Using the physical activity questionnaire, three categories of physical activity were established: vigorous, moderate and less than moderate. Participants categorized as “vigorous” were those who answered “Yes” to the questions about vigorous work or recreational activities. Likewise, participants who replied “Yes” when asked about moderate work or recreational activities were categorized as “moderate”; others were classified as “less than moderate”. “Hypertension” diagnosis was made using information from a self-reported questionnaire or measurements of systolic blood pressure ≥ 140 mmHg and/or diastolic blood pressure ≥ 90 mmHg [21]. “Diabetes” was diagnosed based on: (1) self-reported diagnosis in questionnaires; (2) use of insulin or diabetes medications; (3) plasma glucose level measured by 2-hour oral glucose tolerance test (OGTT) ≥ 200 mg/dL; (4) fasting glucose level ≥ 126 mg/dL; or (5) glycated hemoglobin A1c (HbA1c) ≥ 6.5% [22]. Non-steroidal anti-inflammatory drugs (NSAIDs) and β-blockers were selected to represent the medication covariates in this study because they have the potential to induce and exacerbate psoriasis and are commonly used in the treatment of psoriasis-related comorbidities [23, 24].

### Statistical analysis

The Centers for Disease Control and Prevention (CDC) guidelines were followed in conducting all statistical analyses and a suitable NHANES sampling weight was used for the complex sampling survey design in the analyses [19]. For continuous variables, the mean (95% CI) was used, while for categorical variables, the presentation was as a percentage (95% CI). Since LAP has a non-normal distribution, a natural log transformation was performed to convert it to a normal distribution, denoted “ln LAP”. The study employed two methods to assess differences between groups based on psoriasis status: for continuous variables, weighted linear regression was used, while for categorical variables, the weighted chi-square test was used.

Weighted multivariate logistic regression models were used to investigate the relationship between the LAP index and psoriasis. Covariates were not adjusted in the crude model (Model 1). The minimally adjusted model (Model 2) was adjusted for age, sex and race. The fully adjusted model (Model 3) was adjusted for age, sex, race, education, marital status, PIR, smoking, alcohol intake, physical activity, TC, hypertension, diabetes, NSAIDs and β-blockers. Furthermore, ln LAP was considered as a categorical variable by quartile. To investigate the non-linear correlations between ln LAP and psoriasis, smoothed curve fitting by the generalized additive model was employed. Finally, stratification and interaction analyses were carried out by age, sex, smoking, drinking status, physical activity, hypertension and diabetes. This study used two-sided statistical testing, with ***P*** values < 0.05 indicating statistical significance. R (version 4.1.1) or Empowerstats (version 4.1) were used for all analyses.

## Results

### Characteristics of study participants

This analysis comprised 9,781 participants who were 20 years of age or older. The mean (95% CI) age was 45.16 (44.59, 45.72) years, 49.56% were male and 68.56% were non-Hispanic White. The differences between participants with or without psoriasis are listed in Table 1. Of these, 269 participants (3.08%, 2.63–3.62) had psoriasis and 9,512 (96.92%, 96.38–97.37) did not have psoriasis. Based on the weighted analyses, significant differences (*P* < 0.05) were found between the two groups in terms of age, race, smoking, hypertension, obesity-related parameters (BMI, WC and LAP index), NSAIDs and β-blockers. Psoriasis participants were older and more obese compared with non-psoriasis participants. Additionally, the psoriasis population had higher rates of non-Hispanic White, former smokers, hypertension and NSAIDs or β-blockers usage.

**Table 1:** Basic characteristics of participants by psoriasis in NHANES 2003–2006 and 2009–2014, weighted

Characteristics	Total (*N* = 9,781)	Non-psoriasis (*n* = 9,512)	Psoriasis (*n* = 269)	*P*-value
Age (years)	45.16 (44.59, 45.72)	45.07 (44.50, 45.64)	47.88 (45.53, 50.23)	**0.0214**
PIR	2.94 (2.85, 3.04)	2.94 (2.85, 3.03)	3.08 (2.82, 3.34)	0.2618
BMI (kg/m^2^)	28.75 (28.54, 28.95)	28.71 (28.49, 28.92)	30.07 (29.06, 31.07)	**0.0106**
WC (cm)	98.45 (97.92, 98.98)	98.32 (97.78, 98.86)	102.49 (100.15, 104.82)	**0.0009**
LAP	58.29 (56.21, 60.37)	57.99 (55.90, 60.08)	67.61 (58.15, 77.08)	**0.0477**
Ln LAP	3.70 (3.67, 3.73)	3.70 (3.67, 3.73)	3.91 (3.80, 4.02)	**0.0003**
TC (mmol/L)	5.02 (4.99, 5.05)	5.02 (4.99, 5.05)	5.06 (4.92, 5.21)	0.5427
TG (mmol/L)	1.46 (1.42, 1.50)	1.46 (1.42, 1.50)	1.53 (1.40, 1.67)	0.2870
Sex, %				0.6904
Male	49.56 (48.49, 50.64)	49.61 (48.54, 50.68)	48.17 (41.03, 55.38)	
Female	50.44 (49.36, 51.51)	50.39 (49.32, 51.46)	51.83 (44.62, 58.97)	
Race, %				**< 0.0001**
Mexican American	8.81 (7.30, 10.60)	8.97 (7.44, 10.78)	3.80 (2.34, 6.13)	
Other Hispanic	5.23 (4.14, 6.60)	5.29 (4.18, 6.67)	3.50 (1.96, 6.18)	
Non-Hispanic White	68.56 (65.45, 71.52)	68.16 (65.00, 71.16)	81.34 (75.93, 85.75)	
Non-Hispanic Black	10.52 (9.22, 11.98)	10.66 (9.34, 12.15)	6.23 (4.07, 9.41)	
Other race	6.87 (5.98, 7.88)	6.92 (6.03, 7.95)	5.13 (2.97, 8.74)	
Education level, %				0.2812
Less than high school	16.98 (15.51, 18.56)	17.00 (15.56, 18.55)	16.39 (11.44, 22.91)	
High school	22.20 (20.75, 23.72)	22.33 (20.88, 23.85)	18.02 (13.25, 24.03)	
More than high school	60.82 (58.56, 63.03)	60.67 (58.46, 62.83)	65.60 (57.50, 72.88)	
Marital status, %				0.8682
Cohabitation	64.72 (63.21, 66.20)	64.70 (63.16, 66.21)	65.25 (58.52, 71.43)	
Solitude	35.28 (33.80, 36.79)	35.30 (33.79, 36.84)	34.75 (28.57, 41.48)	
Smoking status				**0.0042**
Never	54.62 (52.88, 56.34)	54.84 (53.11, 56.55)	47.81 (40.65, 55.06)	
Former	22.80 (21.46, 24.20)	22.50 (21.17, 23.89)	32.34 (26.26, 39.08)	
Current	22.58 (21.05, 24.18)	22.66 (21.15, 24.25)	19.85 (14.38, 26.75)	
Drinking status				0.4707
Yes	73.12 (71.39, 74.79)	73.00 (71.24, 74.68)	77.14 (69.58, 83.27)	
No	20.20 (18.65, 21.84)	20.26 (18.68, 21.94)	18.14 (12.68, 25.27)	
Unclear	6.68 (5.90, 7.56)	6.74 (5.95, 7.64)	4.73 (2.18, 9.93)	
Physical activity				0.5095
Vigorous	38.67 (37.15, 40.21)	38.54 (37.00, 40.10)	42.79 (35.09, 50.85)	
Moderate	32.24 (30.99, 33.51)	32.28 (31.00, 33.59)	30.78 (24.52, 37.82)	
Less than moderate	29.09 (27.68, 30.54)	29.18 (27.72, 30.68)	26.44 (20.55, 33.30)	
Hypertension				**0.0014**
Yes	37.40 (35.71, 39.14)	37.00 (35.28, 38.76)	50.08 (41.78, 58.37)	
No	62.60 (60.86, 64.29)	63.00 (61.24, 64.72)	49.92 (41.63, 58.22)	
Diabetes				0.9398
Yes	12.99 (11.92, 14.13)	12.98 (11.93, 14.12)	13.15 (9.21, 18.43)	
No	87.01 (85.87, 88.08)	87.02 (85.88, 88.07)	86.85 (81.57, 90.79)	
NSAIDs				**0.0003**
Yes	6.43 (5.66, 7.29)	6.24 (5.51, 7.07)	12.24 (8.27, 17.74)	
No	93.57 (92.71, 94.34)	93.76 (92.93, 94.49)	87.76 (82.26, 91.73)	
β-blockers				**< 0.0001**
Yes	8.89 (8.06, 9.79)	8.63 (7.80, 9.54)	16.86 (12.52, 22.33)	
No	91.11 (90.21, 91.94)	91.37 (90.46, 92.20)	83.14 (77.67, 87.48)	

Weighted mean (95% CI) for continuous variables, the ***P***-value was calculated by weighted linear regression; weighted percentage (95% CI) for categorical variables, the ***P***-value was calculated by weighted chi-square test. Abbreviations: PIR, poverty-to-income ratio; BMI, body mass index; WC, waist circumference; ln LAP, natural log transformation of LAP. TC: total cholesterol; TG: triglycerides; NSAIDs, non-steroidal anti-inflammatory drugs

### Association between the LAP index and psoriasis

**Fig. 2:**
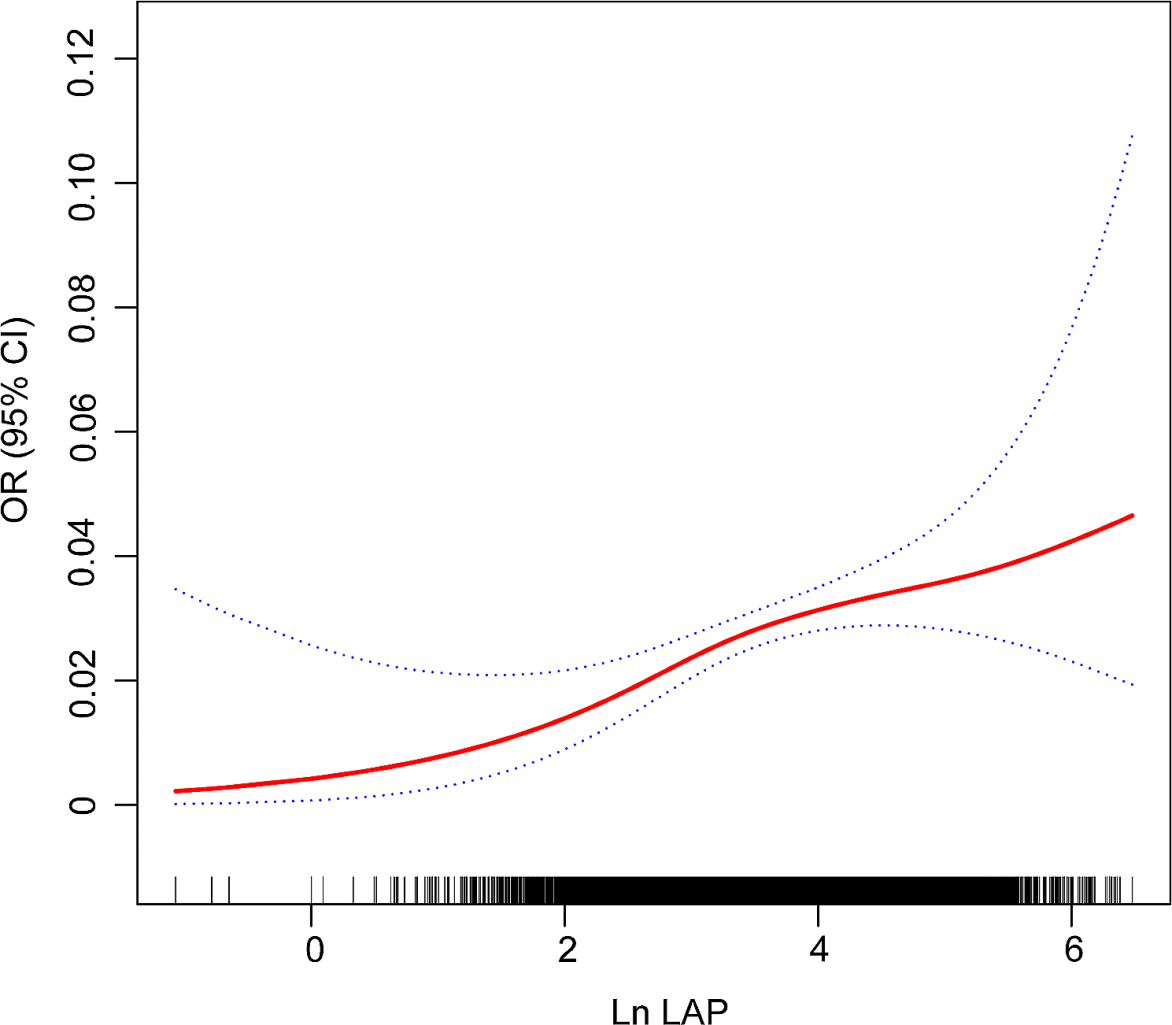
Nonlinear positive association between ln LAP and psoriasis (***P*** for nonlinearity = 0.0133). The solid red line represents the smooth curve fit between variables. The area between two blue dashed lines indicates the fitted 95% confidence interval

The correlation between ln LAP and psoriasis is displayed in Table 2. The results showed that higher ln LAP values were correlated with a higher psoriasis prevalence in Models 1–3. After full adjustment, the probability of having psoriasis increased by 27% for every unit of elevation in ln LAP (OR 1.27, 95% CI 1.06–1.52, *P* = 0.0108). Next, ln LAP was treated as a categorical variable (quartiles) and a sensitivity analysis was performed. In Model 3, the highest ln LAP quartile (Q4) demonstrated a statistically significant 83% increase in the likelihood of psoriasis compared to the lowest quartile (Q1) (OR 1.83, 95% CI 1.08–3.11, *P* = 0.0293). Furthermore, the ***P*** for trend = 0.0277 suggested that psoriasis tended to be more common when the LAP index increased. Smoothed curve fitting by the generalized additive model further displayed a nonlinear positive relationship between ln LAP and psoriasis (*P* = 0.0133; Fig. 2).

**Table 2:** Association between ln LAP and psoriasis in NHANES 2003–2006 and 2009–2014, weighted

	Model 1 OR (95% CI) *P*-value	Model 2 OR (95% CI) *P*-value	Model 3 OR (95% CI) *P*-value
**Continuous ln LAP**	1.33 (1.14, 1.55) **0.0005**	1.29 (1.11, 1.51) **0.0018**	1.27 (1.06, 1.52) **0.0108**
**Quartiles of ln LAP**			
Q1 (−1.069–3.151)	Reference	Reference	Reference
Q2 (3.152–3.720)	1.83 (1.14, 2.93) **0.0141**	1.79 (1.10, 2.89) **0.0210**	1.76 (1.09, 2.85) **0.0245**
Q3 (3.721–4.285)	2.22 (1.39, 3.57) **0.0014**	2.16 (1.34, 3.47) **0.0023**	2.04 (1.23, 3.40) **0.0080**
Q4 (4.286–6.479)	2.06 (1.27, 3.35) **0.0046**	1.95 (1.19, 3.19) **0.0096**	1.83 (1.08, 3.11) **0.0293**
***P*** for trend	**0.0020**	**0.0054**	**0.0277**

Model 1: no covariates were adjusted. Model 2: age, sex and race were adjusted. Model 3: age, sex, race, education level, marital status, PIR, smoking status, drinking status, physical activity, total cholesterol, hypertension, diabetes, NSAIDs and β-blockers were adjusted. In sensitivity analysis, ln LAP was converted from a continuous variable to a categorical variable (quartiles). Abbreviations: OR, odds ratio; 95% CI, 95% confidence interval; Q, quartile

### Subgroup analyses

To evaluate the robustness of the correlation between ln LAP and psoriasis, subgroup analysis and interaction tests were performed (Table 3). The results revealed that the relationship was inconsistent between subgroups. Specifically, there was a significant positive correlation for male participants who were younger than 40, participants who had never smoked, non-drinkers, participants with moderate or less activity, and non-hypertension and non-diabetes participants. The interaction term only detected an effect of drinking status on the correlation between ln LAP and psoriasis (***P*** for interaction = 0.0313). However, no interaction was detected for age, sex, smoking status, physical activity, hypertension or diabetes (all ***P*** for interaction > 0.05). Overall, the results might be most applicable for young males, non-smokers and non-drinkers, participants who engage in little exercise and participants without hypertension or diabetes comorbidities.

**Table 3:** Association between ln LAP and psoriasis in subgroups

Subgroups	*N*	OR (95% CI)	*P*-value	*P* for interaction
**Age (years)**				0.1546
< 40	3,685	1.53 (1.17, 2.02)	**0.0022**	
40–59	3,750	1.09 (0.85, 1.39)	0.5005	
≥ 60	2,346	1.36 (0.95, 1.95)	0.0903	
**Sex**				0.7939
Male	4,852	1.29 (1.04, 1.62)	**0.0232**	
Female	4,929	1.24 (0.99, 1.56)	0.0611	
**Smoking status**				0.1548
Never	5,420	1.55 (1.22, 1.97)	**0.0004**	
Former	2,177	1.13 (0.83, 1.53)	0.4385	
Current	2,184	1.13 (0.82, 1.55)	0.4451	
**Drinking status**				**0.0313**
Yes	6,628	1.15 (0.95, 1.40)	0.1469	
No	2,368	1.99 (1.37, 2.90)	**0.0003**	
Unclear	785	1.30 (0.60, 2.81)	0.5064	
**Physical activity**				0.6146
Vigorous	3,445	1.16 (0.88, 1.52)	0.2840	
Moderate	3,078	1.37 (1.02, 1.84)	**0.0355**	
Less than moderate	3,258	1.38 (1.02, 1.88)	**0.0396**	
**Hypertension**				0.3840
Yes	3,984	1.22 (0.96, 1.54)	0.1014	
No	5,797	1.40 (1.11, 1.77)	**0.0042**	
**Diabetes**				0.1701
Yes	1,693	1.01 (0.67, 1.52)	0.9547	
No	8,088	1.39 (1.15, 1.67)	**0.0005**	

All covariates (as in Model 3) were adjusted except the stratification variable itself

## Discussion

This nationally representative cross-sectional study enrolled 9,781 participants to evaluate the association between the LAP index and adult psoriasis. It was found that ln LAP and psoriasis were nonlinearly positively correlated among U.S. non-institutionalized civilians. This correlation was steady in subgroups stratified by age, sex, smoking status, physical activity, hypertension and diabetes, but was more suitable for men who were younger than 40, participants who had never smoked and non-drinkers, participants who engaged in moderate or less activity, and non-hypertension and non-diabetes participants. The results indicated that LAP was associated with psoriasis at an early stage of onset and in the absence of comorbidities, so it can be hypothesized that LAP has significant implications for the early detection of psoriasis in high-risk individuals.

This is believed to be the first large-scale investigation examining the possible link between LAP and psoriasis. A higher risk of acquiring psoriasis has been connected to several obesity-related indicators—according to prior research—including BMI, WC, waist-to-hip ratio (WHR) and weight change [25, 26]. In a dose-response meta-analysis and systematic review of the prospective study, a relative risk of 1.24 for every 10 cm rise in WC, 1.19 for every 5 unit rise in BMI, 1.37 for every 0.1 unit rise in WHR and 1.11 for a 5 kg weight gain was found [25]. Han et al. conducted a large-sample prospective study with 399,461 psoriasis patients in Korea, finding a higher prevalence of psoriasis in those with a BMI > 30 than those with a normal BMI (HR 1.118, 95% CI 1.100–1.137). Additionally, WC was dose-dependently correlated with psoriasis risk after adjusting for covariates including BMI [26]. Consistent with previous findings, the present study also found that psoriasis patients had significantly higher BMI and WC values than normal participants, especially WC (Table 1).

LAP incorporates another blood indicator related to lipid metabolism—TG. Several studies have confirmed that TG is elevated in adolescents and adults with psoriasis [18, 27–29]. Koebnick et al. conducted a cross-sectional study with 133,270 adolescent participants (439 with psoriasis) and observed that serum TG was higher in psoriasis patients than in adolescents without psoriasis, independent of obesity [27]. To date, there have only been two small-sample case-control studies focused on the correlation between LAP and psoriasis. Ganguly et al. performed one clinical study with 40 chronic plaque psoriasis patients in 2018, which found that psoriasis patients exhibited notably elevated LAP scores in contrast to the control cohort, and that were positively correlated with psoriasis severity subgroups [17]. Another study by Ataseven et al. in 2021 revealed that the LAP index of psoriasis patients was substantially higher than that of healthy controls [18]. The findings of the present study, which was based on a larger sample, validated previous results wherein elevated LAP was correlated with a higher probability of psoriasis. It also identified a more applicable population, that is males younger than 40 years of age, participants who had never smoked, non-drinkers, those with low activity levels, and those with no comorbidities.

For the mechanisms associated with obesity and psoriasis, prior research has demonstrated that both obesity and psoriasis indicate pro-inflammatory states, and there is a significant overlap in the immune mechanisms of these two diseases [30]. In addition to serving as an organ for storing lipids, adipose tissue is an active secretory endocrine organ that generates a variety of pro-inflammatory cytokines and adipokines [31]. Adipose tissue includes mature adipocytes and the stromal vascular fraction (SVF), with the SVF containing multiple cellular components, such as mesenchymal stem cells, vascular endothelial cells, macrophages, nerve cells, T-cells and B-cells. Experiments in mice have revealed that macrophages and related gene expression were elevated in the SVF of obese animals [32]. Activated macrophages secrete inflammatory cytokines such as TNF and IL-6, which are known to make psoriasis symptoms worse [33]. Lande et al. found increased levels of cathelicidin from intradermal adipocytes in lesions of psoriasis individuals, with its pro-inflammatory effects potentially contributing to the psoriasis etiology [34]. Additionally, memory T-cells stored in white adipose tissue that are activated during adaptive immunity may lead to an inflammatory response in psoriasis [30, 35]. The three adipokines in psoriasis that have been investigated the most are leptin, resistin, and adiponectin [36]. Leptin and resistin are both pro-inflammatory adipokines, whereas adiponectin inhibits TNF-α to have an anti-inflammatory effect [37]. High levels of leptin were linked to both the severity of obesity and psoriasis, according to a meta-analysis of 26 studies conducted by Kyriakou et al. [38]. Leptin promotes the elevated production of Th 1 cells and IL-17 A, which may be related to the pathogenesis of psoriasis [39]. Resistin has also been found to be correlated with the onset and progression of psoriasis, which may be related to its pro-inflammatory effects resulting from elevated cytokine production, particularly TNF-α, IL-6, and IL-12 [40–42]. On the contrary, psoriasis patients were found to have lower levels of adiponectin, an anti-inflammatory adipokine, in comparison to normal controls [38, 43]. The reciprocal inhibition of adiponectin and TNF-α could explain the decreased adiponectin in psoriasis patients [44, 45]. On the other hand, experiments in mice suggested that adiponectin deficiency led to the over-infiltration of IL-17-producing dermal γδ-T cells, which exacerbated psoriasis-like skin inflammation [46].

Biologics are becoming a common therapeutic option for psoriasis. Several studies have found that biological treatments may improve MetS-related markers in psoriatic patients, such as lipid, uric acid and inflammatory parameters [47, 48]. Hagino et al. conducted a retrospective study with 165 psoriatic patients and observed that TNF-α inhibitors may improve hyperuricemia and dyslipidemia [47]. In a prospective study, Piros É.A. et al. treated 35 adult patients with severe plaque-type psoriasis with anti-IL-17 antibody, which led to an improvement in high-density lipoprotein-cholesterol and significant decreases in CRP and low-density lipoprotein-cholesterol [48]. These findings demonstrated the close association of psoriasis treatment with metabolic health, and the intricate relationship with lipid accumulation and MetS. It is well known that WC and serum TG are not only diagnostic conditions for MetS but are also closely associated with psoriasis, making LAP a promising indicator for the evaluation of biological treatments in psoriasis.

## Study strengths and limitations

There are several strengths of this study. The relationship between LAP and psoriasis was examined for the first time in a broad cross-sectional investigation, which made the study population nationally representative by taking into account the NHANES sample weighting design. Furthermore, the sufficiently large sample size allowed us to stratify the study population and further validate the stability of the results. In addition, covariates that may influence the relationship between LAP and psoriasis were adjusted to make the results more reliable.

However, there are some inherent limitations in this research. Firstly, self-reported questionnaires may lead to recall bias in psoriasis diagnosis. Secondly, the causal relationship between LAP and psoriasis could not be established in this cross-sectional investigation. Thirdly, as NHANES lacked sufficient information on the severity of psoriasis, it was difficult for us to perform an analysis of the LAP index and psoriasis grading or classification. Fourthly, even though possible covariates and confounding variables were adjusted, there are still some factors that may affect the results of this study. For example, lifestyle factors (such as diets, emotions, and stress levels), genetic predisposition, and the use of various medications in daily life might affect the relationship between LAP and psoriasis. Besides, WC and TG are only available for a single measurement, and the results might be more convincing if there are different stages of data.

## Conclusions

In a large-scale, nationally representative sample, the current study is the first to discover a positive correlation between LAP and psoriasis, revealing the close clinical relevance of lipid metabolism and obesity to psoriasis. The present findings suggest that LAP is a predictor of psoriasis and it is recommended that attention be paid to LAP scores in individuals with high risk of psoriasis (e.g., those with a family history or presenting with ambiguous skin lesions). Abnormal LAP scores can be used to identify potential psoriasis risk, allowing for a comprehensive physical health assessment.

In summary, the results of this study offer a valuable instrument for the early detection of psoriasis, especially in young males, participants who never smoked, non-drinkers, participants who exercised little, as well as non-hypertensive and non-diabetic populations. This would support personalized treatment and monitoring, thereby enhancing the refined management of psoriasis.

### Electronic supplementary material

Below is the link to the electronic supplementary material.

Supplementary Material 1

Supplementary Material 2

Supplementary Material 3

## Data Availability

The datasets supporting the conclusions of this article are available at https://www.cdc.gov/nchs/nhanes/.

## Abbreviations

LAP: Lipid accumulation product
ln LAP: Natural log transformation of LAP
NHANES: National Health and Nutrition Examination Survey
NCHS: National Center for Health Statistics
U.S.: United States
MetS: Metabolic syndrome
NAFLD: Non-alcoholic fatty liver disease
PIR: Poverty-to-income ratio
BMI: Body mass index
WC: Waist circumference
TG: Triglycerides
TC: Total cholesterol
NSAIDs: Non-steroidal anti-inflammatory drugs
