# Supplementary material for: Association between lipid accumulation product and psoriasis among adults: a nationally representative cross-sectional study
Source: Lipids Health Dis. 2024 May 17;23:143. doi: 10.1186/s12944-024-02123-y (PMC11100150; doi:10.1186/s12944-024-02123-y)
Supplement: Supplementary file 1 — Supplementary Material 1 [file 12944_2024_2123_MOESM1_ESM.pdf]

766347164719775744.docx

by Caiyun Zhang

Submission date: 16-Apr-2024 09:43PM (UTC-0700)
Submission ID: 2352446573
File name: 766347164719775744.docx (122.32K)
Word count: 3343
Character count: 19687

Association between lipid accumulation product and psoriasis among adults: A nationally representative cross-sectional study

Abstract

Background: LAP (lipid accumulation product) is an accessible and relatively comprehensive assessment of obesity that represents both anatomical and physiological lipid accumulation. Obesity and psoriasis are potentially related, according to earlier research. Investigating the relationship between adult psoriasis and the LAP index is the goal of this study.

Methods: This is a cross-sectional study based on data from the National Health and Nutrition Examination Survey (NHANES) 2003-2006 and 2009-2014. The association between LAP and psoriasis was examined using multivariate logistic regression and smoothed curve fitting. To verify whether this relationship was stable across populations, this study performed subgroup analyses and interaction tests.

Results: The LAP index showed a positive correlation with psoriasis in 9,781 adult participants who were 20 years of age or older. A 27% elevated probability of psoriasis was linked to every unit increase in ln LAP in the fully adjusted model (Model 3; OR 1.27; 95% CI 1.06-1.52). In comparison with participants in the lowest quartile, those in the highest quartile of ln LAP had an 83% greater likelihood of psoriasis (Model 3; OR 1.83; 95% CI 1.08-3.11). This positive correlation was more suitable for young males, who smoke, drink, and exercise less, non-hypertension and non-diabetes population.

Conclusions: According to this research, the LAP index and adult psoriasis are positively correlated, especially in young males without comorbidities. This study proposed that LAP

may serve as a biomarker for early diagnosis and tracking the efficacy of biological treatments in psoriasis.

Keywords: NHANES, Psoriasis, Lipid accumulation product, Obesity

1. Background

Approximately 3.0% of the United States (U.S.) adult population suffers from psoriasis, a prevalent immune-mediated disease[1]. The incidence of psoriasis is comparable in women and men. 18 to 39 and 50 to 69 years are the two peak ages for psoriasis, although it can develop at any age[2]. It is well known that psoriasis not only causes extensive, recurrent patches and plaques of the skin, but may also be accompanied by multiple metabolism-related comorbidities, such as obesity, hypertension, diabetes, dyslipidemia, non-alcoholic fatty liver disease (NAFLD) and metabolic syndrome (MetS)[3, 4]. Psoriasis has resulted in a significant socioeconomic burden due to its incurability and potential involvement of multiple organ systems[5, 6].

The prevalence of obesity has steadily climbed globally over the past few years, posing a serious threat to health[7]. Obesity is strongly linked to the prevalence of metabolism-related diseases, such as type 2 diabetes, hypertension, obstructive sleep apnea, NAFLD and cardiovascular disease[8]. Currently, the commonly used clinical indicators for obesity assessment are body mass index (BMI) and waist circumference (WC). BMI represents overall obesity but cannot assess different body components, such as bone density, muscle mass and distribution of fat, so some researchers believe that its role is relatively crude and controversial in assessing the risk of some diseases and mortality[9-11]. WC is used to assess the degree of

abdominal obesity (or central obesity), which causes metabolic disorders, leading to diabetes, NAFLD, MetS and cardiovascular disease[8, 12]. However, WC couldn't distinguish between visceral and subcutaneous adipose tissue, and the former indicates ectopic fat accumulation, which causes organ dysfunction and insulin resistance[8]. LAP (lipid accumulation product) uses WC and fasting triglycerides (TG) concentration to characterize lipid overaccumulation, which can reflect the combined anatomic and physiologic changes in adults[13]. Several research works have suggested that LAP could be a potential indicator for cardiovascular disease, type 2 diabetes, insulin resistance, NAFLD and MetS[13-16].

According to two single-center case-control studies conducted to date, psoriasis patients had a considerably higher LAP index than healthy individuals[17, 18]. Nevertheless, no prior research has looked into the connection between LAP and psoriasis in a population that is nationally representative. The main objective of this research is to explore whether LAP and the prevalence of psoriasis are related, using the 2003-2006 and 2009-2014 National Health and Nutrition Examination Survey (NHANES) datasets. It is hypothesized that increased LAP scores may be associated with a high prevalence of psoriasis. By revealing the link between lipid metabolism, obesity and psoriasis, this study will help to understand psoriasis as a systemic metabolic disorder and its association with other metabolism-related diseases. For clinical practice, LAP may be potentially evaluable for psoriasis diagnosis and chronic disease management.

2. Materials and methods

2.1 Data sources and study population

NHANES, which polls about 5,000 individuals annually from all over the U.S., provided the data for this study. In this project, the nutritional status and general health of the non-institutionalized U.S. population are evaluated, which is being performed by the National Center for Health Statistics (NCHS). Because the study design employed a stratified multistage probability sampling process, it is highly representative[19]. The study's whole set of data is openly and freely accessible at https://www.cdc.gov/nchs/nhanes/.

Five NHANES survey cycles, from 2003 to 2006 and 2009 to 2014, served as the basis for this analysis since only these 5 cycles contained data on both psoriasis status and LAP. The following is a list of the exclusion criteria: (1) participants younger than 20 years of age; (2) participants with missing information to define psoriasis; (3) participants with incomplete data to calculate LAP; (4) pregnant women; (5) participants with missing data on necessary covariates (including 10 on education level, 4 on marital status, 5 on smoking status, 2 on blood pressure information). Ultimately, this study comprised 9,781 participants in total (Figure 1).

2.2 Exposure and outcome definitions

Anthropometric assessments are carried out by trained health technicians at the MEC. Specifically, WC is measured with a tape measure at the upper edge of the iliac crest in centimeters (cm). The following formulas are used to calculate the LAP score: [WC (cm) - 58] × [TG (mmol/L)] for women and [WC (cm) - 65] × [TG (mmol/L)] for men. According to the original protocol, men with WC of 65 cm or less, women with WC of 58 cm or less, and all participants with serum TG concentrations > 15 mmol/L were excluded[13].

Psoriasis diagnosis was made using a self-reported questionnaire, and for different cycles was the DEQ053 or MCQ070. Specifically, participants were asked that "Have you ever been told by a doctor or other health care professional that you had psoriasis?" The reply options were "Yes" and "No". The reliability of self-reported psoriasis conditions has been supported by previous research[20].

2.3 Covariates

This analysis considered the following covariates that could have an impact on psoriasis: age (years), sex (male/female), race (Mexican American, other Hispanic, Non-Hispanic White, Non-Hispanic Black and other race), education level (less than high school, high school and more than high school), marital status (cohabitation/solitude), poverty-to-income ratio (PIR), smoking status, drinking status, physical activity, total cholesterol (TC, mmol/L), hypertension, diabetes, and medication status. For subgroup analysis, three age groups were used to classify the study population: < 40 years, $40 \leq$ age < 59 years, and ≥ 60 years. Smoking status was grouped as never smoked (<100 cigarettes in life), former smoker (≥100 cigarettes but has quit), and current smoker (≥100 cigarettes and currently smoking). Alcohol intake status was classified by the question "Had at least 12 alcohol drinks/1 yr?". Using the physical activity questionnaire (PAQ), three categories of physical activity were established: vigorous, moderate, and less than moderate. Participants categorized as "Vigorous" were those who answered "Yes" to the questions about vigorous work or recreational activities. Likewise, participants who replied "Yes" when asked about moderate work or recreational activities were categorized as "Moderate", others were classified as "Less than moderate". "Hypertension" diagnosis was

made using information from a self-reported questionnaire, or measurements of diastolic blood pressure ≥ 90 mmHg and/or systolic blood pressure ≥ 140 mmHg[21]. “Diabetes” was diagnosed based on: (1) Self-reported diagnosis in questionnaires; (2) Use of insulin or diabetes medications; (3) Plasma glucose level measured by 2-hour OGTT ⩾ 200 mg/dL; (4) Fasting glucose level ⩾ 126 mg/dL; (5) Glycated hemoglobin HbA1c ≥ 6.5%[22]. Non-steroidal anti-inflammatory drugs (NSAIDs) and β-blockers were selected to represent the medication covariates in this study, because they have the potential to induce and exacerbate psoriasis, and are commonly used in the treatment of psoriasis-related comorbidities[23, 24].

2.4 Statistical analysis

Centers for Disease Control and Prevention (CDC) guidelines were followed in conducting all statistical analyses, and a suitable NHANES sampling weight was used for the complex sampling survey design in the analyses[19]. For continuous variables, the mean (95% CI) was used, while for categorical variables, the presentation was as a percentage (95% CI). Since LAP has a non-normal distribution, an LN transformation was performed to convert it to a normal distribution, named ln LAP. The study employed two methods to assess differences between groups based on psoriasis status: for continuous variables, use weighted linear regression; while for categorical variables, use weighted chi-square test.

Weighted multivariate logistic regression models were used to investigate the relationship between the LAP index and psoriasis. Covariates were not adjusted in the crude model (Model 1). In minimally adjusted model (Model 2), age, sex and race were adjusted. In fully adjusted model (Model 3), age, sex, race, education, marital status, PIR, smoking, alcohol intake,

physical activity, TC, hypertension, diabetes, NSAIDs and β-blockers were adjusted. Furthermore, ln LAP was considered as a categorical variable by quartile. To investigate the non-linear correlations between ln LAP and psoriasis, the smoothed curve fitting by the generalized additive model was employed. Lastly, stratification and interaction analyses were carried out by age, sex, smoking, drinking status, physical activity, hypertension, and diabetes. This study used two-sided statistical testing, with statistically significant P values < 0.05. Empowerstats (version 4.1) or R (version 4.1.1) were used for all analyses.

3. Results

3.1 Characteristics of study participants

This analysis comprised 9,781 participants who were 20 years of age or older, with a mean (95% CI) age of 45.16 (44.59, 45.72) years, 49.56% male, and 68.56% non-Hispanic white. The differences between participants with or without psoriasis are listed in Table 1. Of these, 269 participants (3.08%; 2.63-3.62) had psoriasis, and 9,512 (96.92%; 96.38-97.37) did not have psoriasis. Based on the weighted analyses, significant differences ($P < 0.05$) were found between the two groups in terms of age, race, smoking, hypertension, obesity-related parameters (BMI, WC and LAP index), NSAIDs and β-blockers. Psoriasis participants were older and fatter compared with non-psoriasis. In addition, the psoriasis population had higher rates of non-Hispanic White, former smokers, hypertension, NSAIDs and β-blockers usage.

3.2 Association between the LAP index and psoriasis

The correlation between ln LAP and psoriasis is displayed in Table 2. The results showed that higher ln LAP was correlated with a higher psoriasis prevalence in the crude model and two

adjusted models. After fully adjusted, the probability of having psoriasis increased by 27% for every unit of elevated ln LAP (OR 1.27; 95% CI 1.06-1.52; P = 0.0108). Next, ln LAP was treated as a categorical variable (quartiles) and sensitivity analysis was performed. In Model 3, the highest ln LAP quartile (Q4) demonstrated a statistically significant 83% increase in the likelihood of psoriasis as compared to the lowest quartile (Q1) (OR 1.83; 95% CI 1.08-3.11; P = 0.0293). Moreover, the P for trend = 0.0277 suggested that psoriasis tended to be more common when the LAP index increased. In addition, smoothed curve fitting by generalized additive model further displayed a nonlinear positive relationship between ln LAP and psoriasis (P = 0.0133) (Figure 2).

3.3 Subgroup analyses

To evaluate the robustness of the correlation between ln LAP and psoriasis, subgroup analysis and interaction tests were performed (Table 3). The results revealed that the relationship was inconsistent between subgroups. Specifically, there was a significant positive correlation in male participants less than 40 years old, never smokers, no drinkers, moderate or less activity participants, non-hypertension and non-diabetes population. The interaction term only detected an effect of drinking status on the correlation between ln LAP and psoriasis (P for interaction = 0.0313). However, no interaction was detected in age, sex, smoking status, physical activity, hypertension and diabetes (all P for interaction > 0.05). Overall, the results might be more appropriate for young males, who smoke, drink and exercise less, with no comorbidities of hypertension and diabetes.

4. Discussion

This nationally representative cross-sectional study enrolled 9,781 participants was to evaluate the association between the LAP index and adult psoriasis. It was found that ln LAP and psoriasis are nonlinearly positively correlated among U.S. non-institutionalized civilians. This correlation was steady in subgroups stratified by age, sex, smoking status, physical activity, hypertension and diabetes, but more suitable for men aged < 40 years, never smokers, no drinkers, moderate or less activity participants, non-hypertension and non-diabetes population. The present results indicated that LAP was associated with psoriasis at an early stage of onset and in the absence of comorbidities, so it can be hypothesized that LAP has significant implications for early detection of psoriasis in high-risk individuals.

As far as we know, this is the first large-scale investigation examining the possible link between LAP and psoriasis. A higher risk of acquiring psoriasis has been connected to several obesity-related indicators, according to prior research, including BMI, WC, waist-to-hip ratio (WHR) and weight change[25, 26]. In a dose-response meta-analysis and systematic review of the prospective study, researchers found a relative risk of 1.24 for every 10-cm rise in WC, 1.19 for every 5-unit rise in BMI, 1.37 for every 0.1-unit rise in WHR, and 1.11 for a 5-kg of weight gain[25]. Han *et al.* conducted a large-sample prospective study with 399,461 psoriasis patients in Korea, they found a higher prevalence in those with a BMI > 30 than those with normal BMI (HR 1.118; 95%CI 1.100-1.137). Meanwhile, WC was dose-dependently correlated with the risk of psoriasis after adjusting for covariates including BMI[26]. Consistent with previous findings, the present study also found that psoriasis patients had significantly higher BMI and WC than normal participants, especially WC (Table 1).

LAP incorporates another blood indicator related to lipid metabolism-TG, and several studies have confirmed that TG was elevated in adolescents and adults with psoriasis[18, 27-29]. Koebnick *et al.* conducted a cross-sectional study with 133,270 adolescent participants (439 with psoriasis) and observed serum TG was higher in psoriasis patients than in adolescents without psoriasis, independent of obesity[27]. To date, there are only two small-sample case-control studies focused on the correlation between LAP and psoriasis. Ganguly *et al.* performed one clinical research with 40 chronic plaque psoriasis patients in 2018, which found that psoriasis patients exhibited a notably elevated LAP score in contrast to the control cohort, and positively correlated with psoriasis severity subgroups[17]. Another study was conducted by Ataseven *et al.* in 2021, they also discovered that psoriasis patients' LAP index was substantially higher than that of healthy controls[18]. The findings of this study validated previous results that elevated LAP is correlated with a higher probability of psoriasis in a larger sample, and identified a more applicable population, that is males younger than 40 years of age, never smokers, no drinkers, low activity level, and with no comorbidities.

For the mechanisms associated with obesity and psoriasis, prior research has demonstrated that both obesity and psoriasis indicate pro-inflammatory states, and there is a significant overlap in the immune mechanisms of these two diseases[30]. In addition to serving as an organ for storing lipids, adipose tissue is an active secretory endocrine organ that generates a variety of pro-inflammatory cytokines and adipokines[31]. Adipose tissue includes mature adipocytes and stromal vascular fraction (SVF), while SVF contains multiple cellular components, such as mesenchymal stem cells, vascular endothelial cells, macrophages, nerve cells, T-cells and

B-cells. Experiments in mice have revealed that the macrophages and related gene expression were elevated in the SVF of obese animals[32]. Activated macrophages secrete inflammatory cytokines such as TNF and IL-6, which are known to make psoriasis symptoms worse[33]. Lande *et al.* found increased levels of cathelicidin by intradermal adipocytes in lesions of psoriasis individuals, and its pro-inflammatory effects might potentially play a role in the psoriasis etiology[34]. In addition, memory T-cells stored in white adipose tissue that are activated during adaptive immunity may lead to inflammatory response in psoriasis[30, 35]. Three adipokines in psoriasis that have been investigated the most are leptin, resistin, and adiponectin[36]. Leptin and resistin are both pro-inflammatory adipokines, whereas adiponectin inhibits TNF-α to have an anti-inflammatory effect[37]. High levels of leptin were linked to both the severity of obesity and psoriasis, according to a meta-analysis of 26 studies conducted by Kyriakou *et al.*[38]. Leptin promotes elevated production of Th 1 cells and IL-17A, which may be related to the pathogenesis of psoriasis[39]. Resistin has also been found to be correlated with the onset and progression of psoriasis, which may be related to its pro-inflammatory effects resulting from elevated cytokine production, particularly TNF-α, IL-6, and IL-12[40-42]. Psoriasis patients had lower levels of adiponectin, an anti-inflammatory adipokine, in comparison to normal controls[38, 43]. The reciprocal inhibition of adiponectin and TNF-α could explain the decreased adiponectin in psoriasis patients[44, 45]. Apart from this, experiments in mice suggested that adiponectin deficiency led to over-infiltration of IL-17-producing dermal γδ-T cells, which exacerbated psoriasis-like skin inflammation[46].
Currently, biologics are becoming a common therapeutic option for psoriasis, and several

studies have found that biological treatments may improve MetS-related markers in psoriatic patients, such as lipid, uric acid and inflammatory parameters[47, 48]. Hagino T. *et al.* conducted a retrospective study with 165 psoriatic patients and observed that TNF-α inhibitors may improve hyperuricemia and dyslipidemia[47]. In a prospective study, Piros É.A. *et al.* treated 35 adult patients with severe plaque-type psoriasis with anti-IL-17 antibody, which led to an improvement in high-density lipoprotein-cholesterol and significant decreases in CRP and low-density lipoprotein-cholesterol[48]. These findings demonstrated the close association of psoriasis treatment with metabolic health, and the intricate relationship with lipid accumulation and MetS. It is well known that WC and serum TG are not only diagnostic conditions for MetS, but also are closely associated with psoriasis, making LAP a promising indicator for the evaluation of biological treatments in psoriasis.

5. Study strengths and limitations

There are several strengths of this study. The relationship between LAP and psoriasis is being examined for the first time in a broad cross-sectional investigation, which made the study population nationally representative by taking into account the NHANES sample weighting design. Furthermore, the sufficiently large sample size allows us to stratify the study population and further validate the stability of the results. In addition, covariates that may influence the relationship between LAP and psoriasis were adjusted to make the results more reliable. However, there are some inherent limitations in this research. Firstly, self-reported questionnaires may lead to recall bias in psoriasis diagnosis. Secondly, the causal relationship between LAP and psoriasis could not be established in this cross-sectional investigation.

Thirdly, as NHANES lacked sufficient information on the severity of psoriasis, it was difficult for us to perform an analysis of the LAP index and psoriasis grading or classification. Fourthly, even though possible covariates and confounding variables were adjusted, there are still some factors that may affect the results of this study. For example, lifestyle factors (such as diets, emotions, and stress levels), genetic predisposition, and the use of various medications in daily life might affect the relationship between LAP and psoriasis. Besides, WC and TG are only available for a single measurement, and the results might be more convincing if there are different stages of data.

6. Conclusions

In a large-scale, nationally representative sample, the current study is the first to discover a positive correlation between LAP and psoriasis, revealing the close clinical relevance of lipid metabolism and obesity to psoriasis. The present findings suggest that LAP is a predictor, and it is recommended that attention be paid to LAP scores in individuals at high risk for psoriasis (e.g., those with a family history, or presenting with ambiguous skin lesions). Abnormal LAP scores can be used to identify potential psoriasis risk, allowing for a comprehensive physical health assessment. To summarize, the results of this study offer a valuable instrument for early detection of psoriasis, especially in young males, those who smoke, drink, and exercise less, and those who are non-hypertensive and non-diabetic, and support personalized treatment and monitoring, thereby enhancing the refined management of psoriasis.

766347164719775744.docx

ORIGINALITY REPORT

12%	10%	8%	1%
SIMILARITY INDEX	INTERNET SOURCES	PUBLICATIONS	STUDENT PAPERS

PRIMARY SOURCES

1	www.researchgate.net<br>Internet Source	2%
2	www.researchsquare.com<br>Internet Source	1%
3	www.frontiersin.org<br>Internet Source	1%
4	Shenjian Chen, Mengqin Luo, Zhiyong Sheng, Rui Zhou, Wenwen Xiang, Wei Huang, Yu Shen. "Association of lipid accumulation product with all-cause and cardiovascular disease mortality: Result from NHANES database", Nutrition, Metabolism and Cardiovascular Diseases, 2023<br>Publication	1%
5	Jie Huang, Jiaheng Han, Rigbat Rozi, Bensheng Fu, Zhengcao Lu, Jiang Liu, Yu Ding. "Association between lipid accumulation products and osteoarthritis among adults in the United States: A cross-sectional study, NHANES 2017-2020", Preventive Medicine, 2024	1%

	Publication	
6	bmcpsychiatry.biomedcentral.com<br>Internet Source	1 %
7	Xiya Zhao, Junqin Li, Xinhua Li. "The association between weight-adjusted waist index and psoriasis in adults: results from NHANES 2009-2014", Research Square Platform LLC, 2023<br>Publication	1 %
8	www.science.gov<br>Internet Source	1 %
9	Li Jiao, Amy Berrington de Gonzalez, Patricia Hartge, Ruth M. Pfeiffer et al. "Body mass index, effect modifiers, and risk of pancreatic cancer: a pooled study of seven prospective cohorts", Cancer Causes & Control, 2010<br>Publication	<1 %
10	iris.uniroma1.it<br>Internet Source	<1 %
11	www.wjgnet.com<br>Internet Source	<1 %
12	www2.mdpi.com<br>Internet Source	<1 %
13	Dan Vata, Bogdan Marian Tarcau, Ioana Adriana Popescu, Ioana Alina Halip et al.	<1 %

"Update on Obesity in Psoriasis Patients", Life, 2023
Publication

14 profiles.wustl.edu
Internet Source
<1%

15 test.diva-portal.org
Internet Source
<1%

16 Xin Gao, Guiyan Wang, Aili Wang, Tan Xu, Weijun Tong, Yonghong Zhang. "Comparison of lipid accumulation product with body mass index as an indicator of hypertension risk among Mongolians in China", Obesity Research & Clinical Practice, 2013
Publication
<1%

17 worldwidescience.org
Internet Source
<1%

18 www.mdpi.com
Internet Source
<1%

19 www.sciforschenonline.org
Internet Source
<1%

20 www.wrc.org.za
Internet Source
<1%

21 Rajiv Khandekar, Ali Jaffer Mohammed. "Coverage of cataract surgery per person and per eye: Review of a community-based
<1%

blindness survey in Oman", Ophthalmic Epidemiology, 2009
Publication

22	Tingting Du, Xuefeng Yu, Jianhua Zhang, Xingxing Sun. "Lipid accumulation product and visceral adiposity index are effective markers for identifying the metabolically obese normal-weight phenotype", Acta Diabetologica, 2015<br>Publication	<1 %
23	academic.oup.com<br>Internet Source	<1 %
24	archpublichealth.biomedcentral.com<br>Internet Source	<1 %
25	www.ncbi.nlm.nih.gov<br>Internet Source	<1 %

Exclude quotes	On	Exclude matches	Off
Exclude bibliography	On		
