# Supplementary material for: Association between lipid accumulation product and psoriasis among adults: a nationally representative cross-sectional study
Source: Lipids Health Dis. 2024 May 17;23:143. doi: 10.1186/s12944-024-02123-y (PMC11100150; doi:10.1186/s12944-024-02123-y)
Supplement: Supplementary file 2 — Supplementary Material 2 [file 12944_2024_2123_MOESM2_ESM.pdf]

NATIVE English Editing

https:www.nativeee.com

Address: 18 East Jiuxianqiao Road,

Chaoyang District, Beijing, China

Phone: +861064125081

19th April, 2024

STATEMENT OF EDITING

This is to certify that the following document has been checked and corrected for proper English language, grammar, punctuation, spelling, and overall style by one or more of the highly-qualified, native English-speaking editors at Native English Editing.

Native English Editing provides editing and proofreading of scientific manuscripts for submission to peer-reviewed journals.

Manuscript title: Association between lipid accumulation product and psoriasis among adults: A nationally representative cross-sectional study

Date Issued: 19th April, 2024

Certificate Verification Key: 2024041756551029

Yours truly,

Graeme Brown

Co-owner Native English Editing

Email: publish@nativeee.com

Contact information of Beijing sales department in China:

Address: 18 East Jiuxianqiao Road, Chaoyang District, Beijing, China

Phone: +861064125081

Fax: +861064125081

Contact information of Australian editorial department:

Address: 42D Melrose Street, Parkdale Vic 3195, Australia

Phone: +61417560758
