# Supplementary material for: Association between lipid accumulation product and psoriasis among adults: a nationally representative cross-sectional study
Source: Lipids Health Dis. 2024 May 17;23:143. doi: 10.1186/s12944-024-02123-y (PMC11100150; doi:10.1186/s12944-024-02123-y)
Supplement: Supplementary file 3 — Supplementary Material 3 [file 12944_2024_2123_MOESM3_ESM.docx]

**Title page**

**Manuscript type: Research**

**Title of the article:** Association between lipid accumulation product and psoriasis among adults: A nationally representative cross-sectional study.

**Authors:** Caiyun Zhang^1^, Xiaoping Dong^2^, Jun Chen^3^*, Fang Liu^2^*

**Departments and institutions:**

1 Department of Medical Cosmetology, Nanjing Hospital of Chinese Medicine Affiliated to Nanjing University of Chinese Medicine, Nanjing, China

2 Department of Dermatology, Jinling Hospital, Affiliated Hospital of Medical School, Nanjing University, Nanjing, China

3 Department of Dermatology, The Fourth Affiliated Hospital of Nanjing Medical University, Nanjing, China

**Corresponding Authors:**

*Fang Liu, Department of Dermatology, Jinling Hospital, Affiliated Hospital of Medical School, Nanjing University, Nanjing, 210002, China. Email: [liufangndyx@163.com](mailto:liufangndyx@163.com).

*Jun Chen, Department of Dermatology, The Fourth Affiliated Hospital of Nanjing Medical University, Nanjing, 210031, China. Email: springchenjun@163.com.
